# New Insights into Binding of G-segment DNA to the Active Site of *Escherichia coli* Topoisomerase III

**DOI:** 10.1038/s41598-026-57834-2

**Published:** 2026-08-14

**Authors:** Kemin Tan, Thirunavukkarasu Annamalai, Lucy Stols, Md Anisur Rahman Bhuiyan, Yuk-Ching Tse-Dinh

**Affiliations:** 1 Structural Biology Center, X-ray Science Division, Advanced Photon Source, Argonne National Laboratory, 9700 S. Cass Avenue, Lemont, IL 60439 USA; 2 Department of Chemistry and Biochemistry, Florida International University, 11200 SW 8 St, Miami, FL 33199 USA; 3 Biomolecular Sciences Institute, Florida International University, 11200 SW 8 St, Miami, FL 33199 USA

**Keywords:** *Escherichia coli* Topoisomerase III, EcTopo3, G-Segment binding, Half-open binding mode, Metal-binding site, Biochemistry, Biophysics, Structural biology

## Abstract

*Escherichia coli* topoisomerase III (EcTopo3) is a type IA topoisomerase that binds single-stranded DNA (ssDNA) during DNA cleavage and strand passage. Here, we report five crystal structures of EcTopo3 in complex with distinct 8-base ssDNA oligonucleotides at 1.85–2.22 Å resolution. These structures reveal a previously unrecognized half-open ssDNA-binding mode. In this mode, EcTopo3 engages only the five 3′-terminal nucleotides of the oligonucleotide within the conserved D4/D1 DNA-binding groove, whereas the D1/D3 binding site near the active center remains closed. All five complex structures—three in the open form and two in the half-open form—clearly show that local base binding within the D4/D1 groove is adaptable and involves both direct and water-mediated contacts, consistent with limited sequence specificity. These findings suggest that ssDNA engagement by EcTopo3 may proceed in a stepwise manner, with partial binding in the D4/D1 groove preceding, or occurring independently of, full opening of the D1/D3 site. The half-open structures also identify a distinct metal-binding site on a glycine-rich loop near the active site, occupied by a metal cation coordinated by backbone carbonyls and conserved water molecules. Together, these results reveal greater conformational and mechanistic flexibility in EcTopo3–ssDNA binding than previously appreciated.

## Introduction

DNA topoisomerases manipulate and regulate genome topology to enable essential cellular functions including replication, transcription and recombination^1,2^. Type I topoisomerases catalyze the topological change by introducing a transient single-stranded break in DNA while type II topoisomerase reactions are catalyzed through transient double-stranded DNA breaks. Type I topoisomerases are further divided into subfamilies based on their structure and mechanism. The type IA topoisomerase subfamily is the only universal topoisomerase found in all domains of life^3–5^. The ability of type IA topoisomerases to pass DNA through a single-strand break may be essential for the untangling of certain intermediates formed during replication and recombination^6^.

The type IA topoisomerase subfamily is the only topoisomerase subfamily that can catalyze topological changes in RNA^7^. Although the RNA topoisomerase activity is most robust and associated with physiological functions for human topoisomerase III beta (hTop3B), topoisomerase IA from other eukaryotes, archaea and bacteria also have detectable RNA topoisomerase activity^8^. It has been hypothesized that RNA genome is present in the Last Universal Common Ancestor (LUCA)^9^. A topoisomerase IA may have function there for binding and manipulation of RNA substrates. The transition of RNA to DNA genome over the course of evolution would then require topoisomerase IA to bind and manipulate DNA substrates to overcome topological barriers. Among the two type IA topoisomerases in *Escherichia coli*, the observable RNA topoisomerase activity is much stronger for topoisomerase III (EcTopo3) than topoisomerase I (EcTopo1)^10^. The active site sequence of EcTopo3 shares greater similarities to the topoisomerase III found in archaea and eukaryotes than EcTopo1^11^. In addition to the N-terminal catalytic domains D1-D4 conserved among all type IA topoisomerases^4,12^, EcTopo3 has only a short C-terminal tail of basic residues^13^ instead of the divergent repeats of ssDNA binding motifs found in the C-terminal domains present in other type IA topoisomerases^14,15^. Unlike the topoisomerase III of higher eukaryotes such as hTop3B^15,16^, EcTopo3 acts alone without interacting protein partners for its catalytic function. These considerations suggest that EcTopo3 would be a good model for exploring how a minimal type IA topoisomerase may bind DNA versus RNA substrates in its catalytic activity.

Structures of EcTopo3 in complex with an 8 base long DNA (5’-CGCAACTT-3’, 8MerD1, Table 1) have been reported earlier from crystals harvested from different crystallization and cryoprotection conditions^17,18^. A closed form, an intermediate form and an open form were observed and believed to represent a sequence of steps to approach a catalytically ready conformation^12^. Though the sequence preference of G-segment (ssDNA) for binding of EcTopo3 appears to be weak^19^, it was not known if other 8 base long DNAs of different sequences could bind in a similar way. It is also intriguing whether an 8-base RNA with the same sequence, except for the T-to-U substitution, can bind EcTopo3 as well.

We initiated our study by trying to obtain co-crystals of EcTopo3 bound to varied 8-base long DNA oligonucleotides as well as RNA oligonucleotides of identical sequences except for thymidine to uracil substitution. The oligonucleotide sequences were designed based on previous DNA^17,18^ and RNA cleavage substrates^20^ of EcTopo3. While we were unsuccessful in obtaining EcTopo3-RNA co-crystals, we obtained novel structures of EcTopo3 bound to different DNA oligonucleotides that indicate some degree of sequence preferences of G-segment binding and suggest how a minimal length of DNA substrate may first bind to EcTopo3 before additional DNA sequence can gain access to the enzyme active site in the overall catalytic mechanism.

## Materials and methods

### Cloning, protein purification and crystallization

The gene fragment encoding EcTopo3 (residues M1–K640) was PCR-amplified and cloned into the T7 expression vector pMCSG28^21^. Cloning of EcTopo3 into pMCSG28 introduced a C-terminal His₆-tag preceded by a TEV protease cleavage site.

For protein expression, the plasmid was transformed into *E. coli* SoluBL21 cells by heat shock. Small scale cultures were prepared in 50 mL of Luria Broth containing 150 ug/mL of ampicillin, grown overnight at 37 °C and diluted 1:100 into 1 L cultures for large scale growth. Cell cultures were grown at 37^o^ C to an OD₆₀₀ 0.8–1.0, cooled for 1 h to 18 °C, induced with 0.4 mM IPTG and grown at 18 °C for 16 h. Cultures were centrifuged at 5,000 x g at 4 °C for 20 min. Cell pellets were resuspended in lysis buffer (50 mM HEPES pH 8.0, 500 mM NaCl, 20 mM imidazole, 10 mM β-mercaptoethanol (BME), and 5% (v/v) glycerol) and lysed by using 10X FastBreak™ Cell Lysis Reagent (Promega) with 10 µg/ml lysozyme and Benzonase^®^ Nuclease. The cell suspension was incubated for 30 min on ice prior to centrifugation for 75 min at 25,000 x g. Soluble fraction was collected and the protein was purified by immobilized metal affinity chromatography (IMAC) using 3mL Ni²⁺-Sepharose (Cytiva) packed in a Flex-Column operated on a Vac-Man vacuum system (Promega). Following equilibration of the column with lysis buffer (50mM HEPES pH 8.0, 500mM NaCl, 20mM imidazole 5% glycerol 10 mM β-mercaptoethanol), the soluble fraction was added to the column and washed with 30 mL of lysis buffer followed by 10 mLof lysis buffer containing 50 mM imidazole. The protein was eluted with 50mM HEPES pH 8.0, 500mM NaCl, 250mM imidazole, 5% glycerol, 10mM β-mercaptoethanol. Eluted protein was incubated with His-tagged TEV protease (1:20 molar ratio of TEV to protein) at 4 °C for 16 h and dialyzed overnight at 4 °C against lysis buffer. A second IMAC column equilibrated with lysis buffer was used to remove the cleaved His-tag and His-tagged TEV protease. The column flow through containing the protein and wash fractions were pooled, concentrated using a 50 kDa concentrator (Merck-Millipore) followed by a buffer exchange into 20 mM HEPES pH 7.5, 150 mM NaCl, 2 mM TCEP.

The purified EcTopo3 (~ 20 mg/mL) was added directly to lyophilized DNA/RNA pellet-containing tubes that came from suppliers (Sigma-Aldrich, IDT) in RNAse-free environment. The molar ratio of EcTopo3 to DNA or RNA oligo in each mixture is about 1 to 1. The details of DNA and RNA oligos used in this study are listed in Table 1. The EcTopo3/DNA and EcTopo3/RNA mixtures were screened for crystallization with a Mosquito nanoliter liquid handler (TTP LabTech) using the sitting drop vapor diffusion methods. Crystallization screening was performed using MCSG1-4 (Anatrace) screens at 16 °C. Each crystallization droplet containing 0.4 µL of each protein and precipitating agent was equilibrated against a reservoir containing 140 µL of precipitating solution. EcTopo3 in complex with ssDNA oligos (including 8MerD1, 8MerD2, 8MerD3, 8MerD7 and 8MerD8) each crystallized under one or two conditions. Representative crystallization conditions included 0.2 M ammonium citrate dibasic pH 5.0, 20% (w/v) PEG3350 (EcTopo3-8MerD1, EcTopo3-8MerD7 and EcTopo3-8MerD8), 0.1 M lithium acetate, 20% (w/v) PEG3350 (EcTopo3-8MerD2); and 0.1 M sodium citrate, 20% (w/v) PEG4000 and 20% (v/v) 2-propanol (EcTopo3-8MerD3).

**Table 1:** ssDNA and RNA oligos used in experiments

Oligo	Sequence	Nucleotide binding position	+ 2	+ 1	− 1	− 2	− 3	− 4	− 5	− 6	− 7
DNA 8MerD1	5’-CGCAACTT-3’		T	T	C	A	A	C	G	C	
DNA 8MerD7	5’-ACTGACTT-3’		T	T	C	A	G	T	C	A	
DNA 8MerD8	5’-CTGAACTT-3’		T	T	C	A	A	G	T	C	
DNA 8MerD9	5’-CCGAACTT-3’										
DNA 8MerD2	5’-AACTGTTG-3’					G	T	T	G	T	
DNA 8MerD3	5’-GCAACTGG-3’					G	G	T	C	A	A
DNA 8MerD4	5’- CGACACTT -3’										
DNA 8MerD5	5’- CGAAGTCT -3’										
DNA 8MerD6	5’-CGATGTTT-3’										
RNA 8MerR1	5’-CGCAACUU-3’										
RNA 8MerR2	5’-AACUGUUG-3’										

In contrast, there was no EcTopo3-RNA complex crystal obtained in this series of experiments. Though crystals formed under multiple EcTopo3-RNA conditions, all crystals were EcTopo3 alone, with no bound RNA. Increasing RNA: EcTopo3 molar ratio also failed to produce EcTopo3-RNA complex crystals; instead, it appears to progressively suppress EcTopo3 self-crystallization.

Before X-ray diffraction data collection, all crystals were harvested, treated with cryoprotectant solutions (25% [v/v] glycerol or ethylene glycol in the corresponding mother liquor), and flash-frozen in liquid nitrogen.

### X-ray diffraction and structural determination

X-ray diffraction data were collected at 100 K from the cryocooled crystals at 24-ID-E and 21-ID-F beamlines of Advanced Photo Source at Argonne National Laboratory and the 17-ID-2 beamline of National Synchrotron Light Source II at Brookhaven National Laboratory. The intensities were integrated, scaled, and merged with the HKL-3000 program suite or Xia2^22^. The EcTopo3-DNA structures were determined by using molecular replacement (MR) method^23^ with the previously determined EcTopo3 complex structure (PDB entry 1I7D) or a complex structure determined first in this study as a search template. The final models were completed following iterative rounds of model rebuilding using Coot^24^ and restrained refinement using Phenix.refine^25^; and were validated with Molprobity^26^ (Table 2).

The apo-form EcTopo3 crystals produced in the EcTopo3-RNA co-crystallization conditions as described earlier diffracted weakly, consistent with previous reports^27^. Because the resulting structures were nearly identical to the published apo EcTopo3 structure (PDB entry 1D6M) and provided no additional information, therefore the apo EcTopo3 structures determined in this study were not deposited in the Protein Data Bank. In the following presentation and discussion, the structure from PDB entry 1D6M is used as the representative apo EcTopo3 structure.

**Table 2:** Summary of crystallographic data.

Data collection statistics	8MerD1	8MerD7	8MerD8	8MerD2	8MerD3
Space group	*C*2	*C*2	*C*2	*P*2_1_	*P*2_1_
Unit cell (Å, °)	*a* = 111.3 *b* = 132.6 *c* = 77.24 *β* = 101.3	*a* = 109.9 *b* = 132.4 *c* = 78.77 *β* = 134.2	*a* = 70.52 *b* = 73.15 *c* = 198.7 *β* = 101.3	*a* = 39.92 *b* = 123.1 *c* = 86.28 *β* = 98.74	*a* = 38.69 *b* = 120.9 *c* = 85.61 *β* = 99.34
MW Da (number of amino acids)	72,122 (640)^1^	72,122 (640)^1^	72,122 (640)^1^	72,122 (640)^1^	72,122 (640)^1^
Mol (AU)	1	1	1	1	1
Wavelength (Å)	0.979	0.979	0.979	0.979	0.979
Resolution (Å)	2.22–55.7	2.20–54.4	2.10–34.4	1.90–61.60	1.85–60.50
Number of unique reflections	39,262 (2897)^2^	40,486 (3006)^3^	47,324 (3478)^4^	63,110 (4699)^5^	64,910 (4763)^6^
R_merg_ (%)	5.8 (104)^2^	9.4 (105.0)^3^	9.2 (76.7)^4^	10.1 (90.6)^5^	9.7 (91.7)^6^
R_pim_ (%)	3.4 (59.4)^2^	4.3 (47.8)^3^	5.8 (50.7)^4^	8.6(79.7)^5^	5.1 (48.1)^6^
CC ½	0.999 (0.688)^2^	0.988 (0.473)^3^	0.997 (0.884)^4^	0.985 (0.618)^5^	0.996 (0.454)^6^
Completeness (%)	98.9 (99.2)^2^	99.1 (98.2)^3^	99.9 (99.6)^4^	99.9 (99.8)^5^	99.6 (98.7)^6^
Redundancy	3.9 (3.9)^2^	5.7 (5.7)^3^	6.7 (6.2)^4^	3.4 (3.4)^5^	4.5 (4.6)^6^
I/(σ)	10.3 (1.0)^2^	8.8 (1.3)^3^	8.6 (1.5)^4^	6.2 (1.2)^5^	8.2 (1.5)^6^
Solvent content (%)	56.6	56.6	56.8	53.5	54.9
Phasing					
Resolution range (Å)	3.00-55.7	3.00-54.4	3.00-34.4	3.00-61.6	3.00-60.50
Correlation coefficient (%)	69.0	53.9	50.5	32.6	58.8
Refinement					
Resolution range (Å)	2.22–55.7	2.20–54.4	2.10–34.4	1.90–61.6	1.85–60.50
Number of reflections	39,113	40,292	47,021	62,849	64,581
Completeness (%)	98.3	98.5	98.9	99.4	97.8
* R*_work_/*R*_free_ (%)	22.1/26.0	21.8/23.6	22.7/26.5	19.1/22.8	17.8/21.2
No. of atoms					
Protein/DNA/HETATM	4,905/158/84	4,911/162/140	4,929/162/166	5,015/104/423	4967/125/434
Bond lengths (Å)	0.003	0.003	0.003	0.009	0.013
Bond angles (deg)	0.584	0.563	0.503	0.925	1.141
B-factors (Å^2^)					
Protein main/side chain, DNA	84.3/85.6, 76.0	64.6/66.5, 77.6	61.1/63.2, 53.8	35.4/39.2, 33.8	29.4/33.1, 40.5
Wilson B-factor (Å^2^)	58.4	46.0	39.4	26.4	24.7
Molprobity validation					
Ramachandran outliers (%)	0.00	0.16	0.00	0.00	0.00
Ramachandran favored (%)	96.44	97.09	97.57	98.38	98.38
Rotamer outliers (%)	1.34	0.19	0.57	0.37	0.56
Clashscore	2.97	3.45	3.92	2.63	4.36
PDB entry	11GX	11YY	11FC	11KB	11LB

^1^Not including C-terminal six vector derived residues, ENLYFQ. ^2^(Last resolution bin, 2.22–2.28 Å). ^3^(Last resolution bin, 2.20–2.26 Å). ^4^(Last resolution bin, 2.10–2.15 Å). ^5^(Last resolution bin, 1.90-1.98 Å). ^6^(Last resolution bin, 1.85–1.90 Å). ^7^Molecular replacement method.

## Results

In this study of EcTopo3 in complex with different 8mer ssDNA oligos, we have determined five complex structures in a resolution limit range from 1.85 Å to 2.22 Å. All structures have been refined, yielding models (M1 to P620) with high stereochemical quality, Table 2. The C-terminal segment of the expression construct, spanning G621 to K640, is not resolved in the structure and is therefore presumed to be disordered. A portion of the arch-like D2 domain exhibits partial disorder in two structures.

**Fig. 1:**
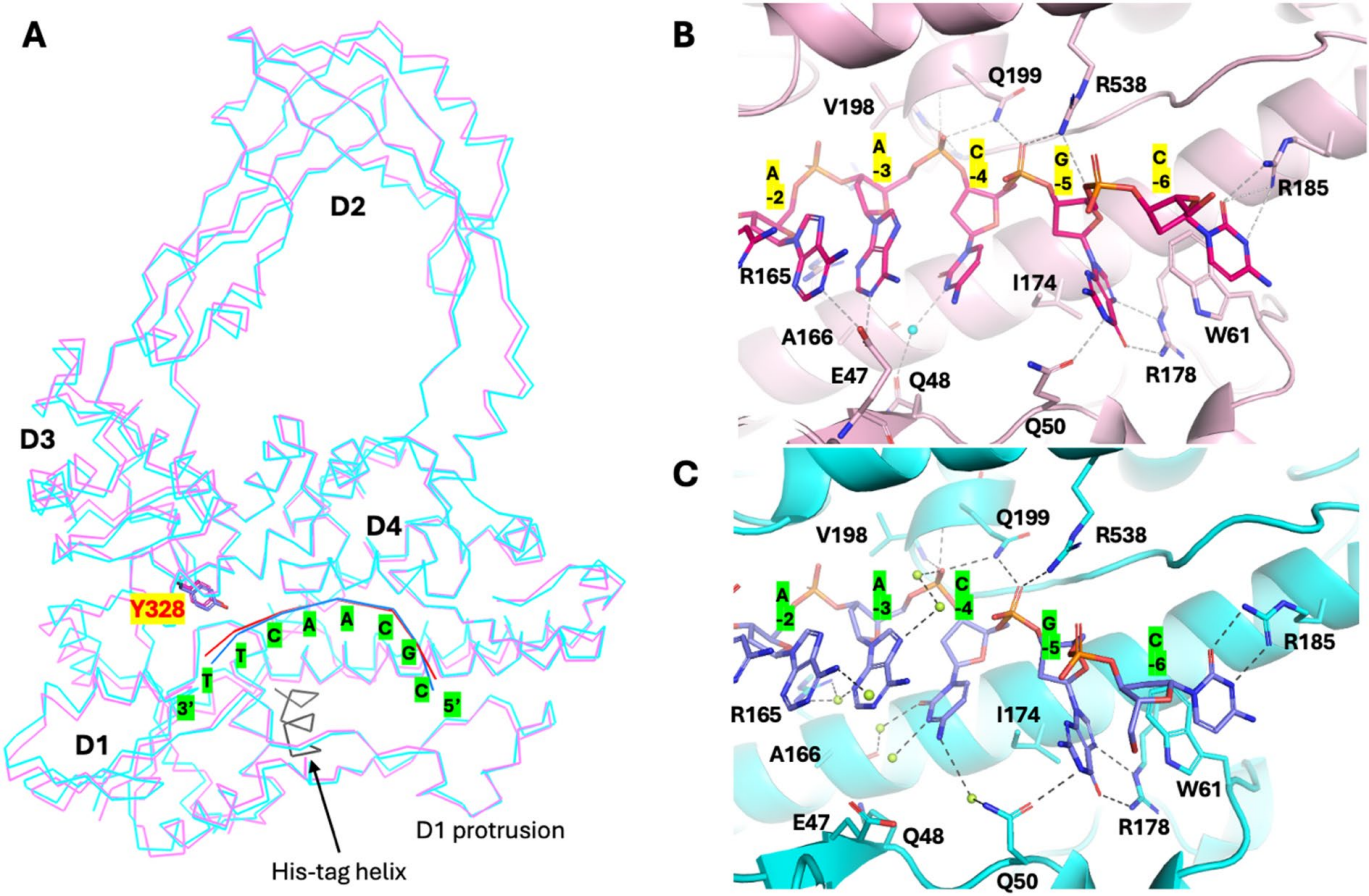
Comparison of two EcTopo3-8MerD1 structures. (**A**) Secondary Structure Matching (SSM) superposition of the EcTopo3-8MerD1 structure determined in this study (enzyme in pink; DNA oligo in red) and the previously reported complex (PDB entry 2O19, chains B-D; enzyme in cyan; DNA oligo in blue; His-tag helix in black). The catalytic residue Y328 is shown in stick representation and highlighted to indicate its position. The protrusion from the D1 domain beneath D4 is labeled. Together, this region of D1 and D4 forms a characteristic DNA-binding groove. The nucleotides of the DNA oligo and the oligo orientation are labeled. (**B**) Enzyme-ssDNA interaction pattern within the D4/D1 DNA-binding groove in the EcTopo3-8MerD1 structure determined in this study. The DNA oligo and enzyme residues involved in the interactions, or otherwise relevant to the figure description, are shown in stick representation. A water molecule is represented by a small green sphere. Each nucleotide and its binding site are indicated in bold black text with yellow shading. Hydrogen bonds and salt bridges are shown as grey dashed lines. (**C**) Enzyme-ssDNA interaction pattern within the D4/D1 DNA-binding groove in EcTopo3-8MerD1 from PDB entry 2O19. Each nucleotide and its binding site are indicated in bold black text with green shading. Water molecules are represented by small lime spheres. Hydrogen bonds and salt bridges are shown as black dashed lines.

Topoisomerase IA binds G-segment (ssDNA) asymmetrically in respect to cleavage site^17^. The ssDNA binding site of EcTopo3 is featured by a ssDNA-binding groove largely within D4 but completed by the protruded part from D1. The D4/D1 DNA-binding groove extends to an interdomain space between D1 and D3 (D1/D3 binding site), Fig. 1A. The two DNA-binding sites are distinct in structure and function as presented below.

### EcTopo3 in complex with 8MerD1, 5’-CGCAACTT-3’

The DNA oligo 8MerD1 was the only oligo used to produce EcTopo3-DNA complexes in previous studies. In the current study, this oligo was used as a control in our co-crystallization of the enzyme with DNA. Its complex crystallized in a low pH (pH = 5.0) condition and its crystal was cryoprotected in 25% glycerol. The condition and treatment were similar to those of the EcTopo3-8MerD1 crystal with structure PDB entry 2O19, among others^18^. The new EcTopo3-8MerD1 structure has a conformation nearly identical to one of two complexes (B-D chains, open conformation) present in the structure, PDB entry 2O19 as shown in Fig. 1A. In an open conformation, the catalytic residue Y328 is positioned to interact with the phosphate group of the scissile nucleotide (Fig. 1A). An alignment of the two open conformational structures using Secondary Structure Matching (SSM)^28^ results in a Root Mean Square Deviation (RMSD) value of 1.03 Å. A simple visual examination reveals a better alignment of the D4 domains while other domains show some relative movement. The arch-like D2 domain in topoisomerase IA structures is well known in mechanistic models for its structural mobility^12,29,30^. In the PDB entry 2O19 structure, the D1 and D3 domains appear to be closer to each other; in particular, D1 appears to be displaced upward. Consequently, the narrower space between the D1 and D3 domains seems to push the two nucleotides at the 3′-end slightly out of the D1/D3 binding site, causing them to adopt a more bent conformation. It is unclear whether these overall conformational differences between two structures of the same protein bound to the same DNA oligonucleotide (8MerD1) arose from different crystallization conditions, molecular packing in crystal, or cryoprotectant treatments. In this particular case, the structure of PDB entry 2O19 contains a short helix derived from the C-terminal expression tag (His-tag helix) that binds between D1 and D4 (Fig. 1A). This crystallographic artifact may have induced unintended structural changes.

Superposition of the D4 domains from the two EcTopo3-8MerD1 structures shows that the domains themselves, as well as the backbones of the five 5′-end nucleotides, are highly well aligned, except for the terminal 5′ nucleotide, which is slightly shifted. The conformational conservation of the D4 domain upon G-segment binding has been noted in previous studies and is discussed further below. Nevertheless, even within this relatively conserved D4/D1 binding groove, the same enzyme can interact with the same substrate in distinct manners (Fig. 1B, C). The interaction pattern beyond the kink between the − 4 and − 5 nucleotides is largely conserved, including the bidentate hydrogen bonds formed between R178 and the guanine at the − 5 position, as well as the stacking interaction among the guanidinium group of R178, the indole ring of W61, and the cytosine base at the − 6 position. In the new EcTopo3-8MerD1 structure (Fig. 1B), E47 forms hydrogen bonds with the adenines at the − 2 and − 3 positions. Q48 interacts with the cytosine at the − 4 position via a water-mediated bridge. In contrast, in the structure of PDB entry 2O19, the bases at the − 2, −3, and − 4 positions appear to be coordinated primarily by water molecules, and no direct contacts are observed between these bases and the enzyme. The substitution of direct hydrogen bonds with water-mediated hydrogen-bonding networks is often associated with increased solvent content and hydration in crystal structures (31, 32).

The solvent content of the crystal of the new EcTopo3–8MerD1 structure is approximately 56.6% (Table 2), whereas that of the crystal of the structure of PDB entry 2O19 is considerably higher, at 68.0%^18^.

### EcTopo3 in complex with 8MerD7 and 8MerD8

Compared with 8MerD1, 8MerD7, 8MerD8, and 8MerD9 (Table 1) contain three to four nucleotide substitutions at the 5′ end, yet only the EcTopo3-8MerD7 and EcTopo3-8MerD8 complexes yielded crystals under the conditions used for EcTopo3-8MerD1. These crystals belonged to the same space group and exhibited comparable unit-cell parameters, solvent contents, and resolution limits (Table 2), indicating a highly similar overall crystal packing environment.

**Fig. 2:**
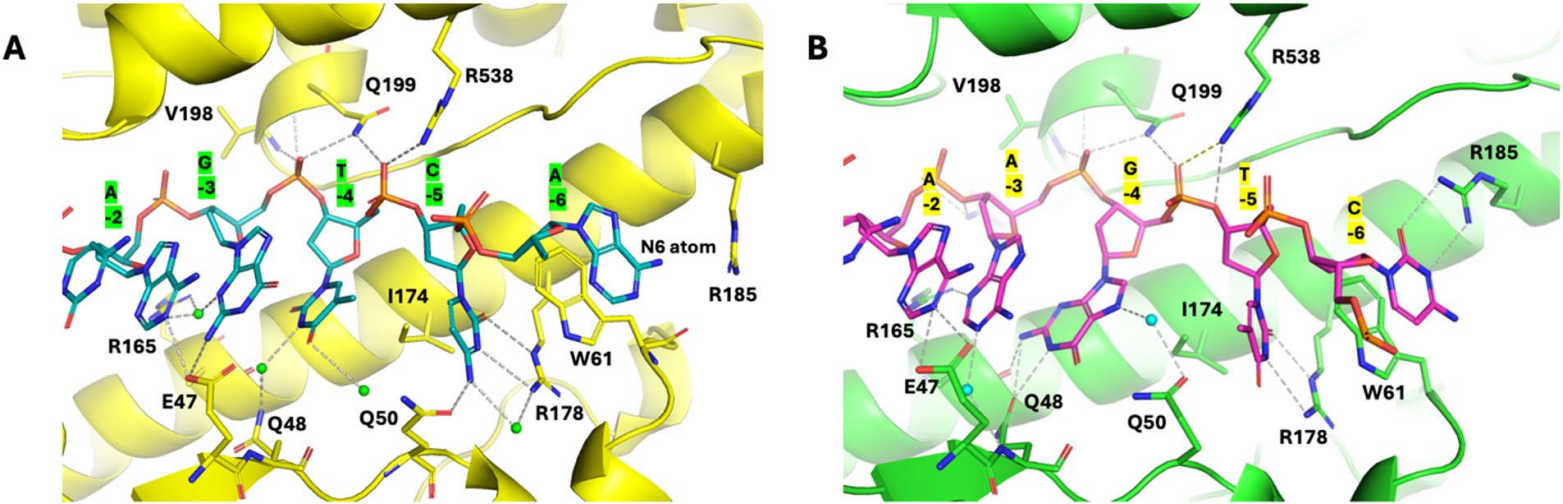
Enzyme-ssDNA interaction patterns within the D4/D1 binding groove of the EcTopo3-8MerD7 and EcTopo3-8MerD8 structures. Aside from the different color schemes, both ribbon diagrams are shown in the same style as those in Fig. 1B and C. (**A**) Interaction pattern within the D4/D1 binding groove of EcTopo3-8MerD7. Notably, R185 does not form hydrogen-bonding interactions with the adenine base at the − 6 position. The N6 atom of the adenine is labeled. (**B**) Interaction pattern within the D4/D1 binding groove of EcTopo3-8MerD8. In addition to stacking with the indole ring of W61, the smaller cytosine base forms bidentate hydrogen bonds with R185, as also observed in the EcTopo3-8MerD1 structure (Fig. 1B and C).

Consistent with this, structural superposition of EcTopo3-8MerD7, EcTopo3-8MerD8, and EcTopo3-8MerD1 gave pairwise RMSD values of less than 0.7 Å, and the phosphate-sugar backbones of the bound ssDNAs were nearly indistinguishable. Thus, despite sequence differences in the bound oligonucleotides, particularly within the D4/D1 binding groove, the enzyme accommodates these substrates with minimal global structural change. A key conserved feature is the bidentate hydrogen bonding between R178 and the base of the nucleotide at the − 5 position (Figs. 1B and 2A and B). In the three structures, this nucleotide is guanine, cytosine, or thymine, respectively, suggesting that the site tolerates multiple bases provided that the required hydrogen-bonding geometry is maintained. Because the − 5 base does not form stacking interactions with neighboring bases or aromatic protein side chains, stabilization at this site likely depends primarily on the bidentate interaction with R178, with Q50 or a local hydrogen-bond network contributing secondarily. In contrast, adenine would be unfavorable at this position because both adenine N6 and the arginine side chain are hydrogen-bond donors, making a favorable interaction unlikely (Fig. 2A). This may represent one structural basis for the ssDNA sequence preference of EcTopo3.

Interestingly, the base at the − 6 position can potentially form an additional bidentate hydrogen-bonding interaction with R185. In the EcTopo3-8MerD7 structure, this interaction is absent because the − 6 nucleotide is adenine. Nevertheless, the adenine base is able to form a favorable stacking interaction with the indole ring of W61, suggesting that the loss of the bidentate hydrogen bonds has only a limited effect on ssDNA binding at this site. At the − 4 position, nucleotide specificity appears to be relatively weak. A purine base can form direct contacts with Q48 (Fig. 2B), whereas a pyrimidine base interacts through one or more water-mediated bridges (Figs. 1B and C and 2A). The sequence preferences at the − 2 and − 3 positions are discussed in the next section in conjunction with the observations described there.

### Half-open EcTopo3-ssDNA binding mode

**Fig. 3:**
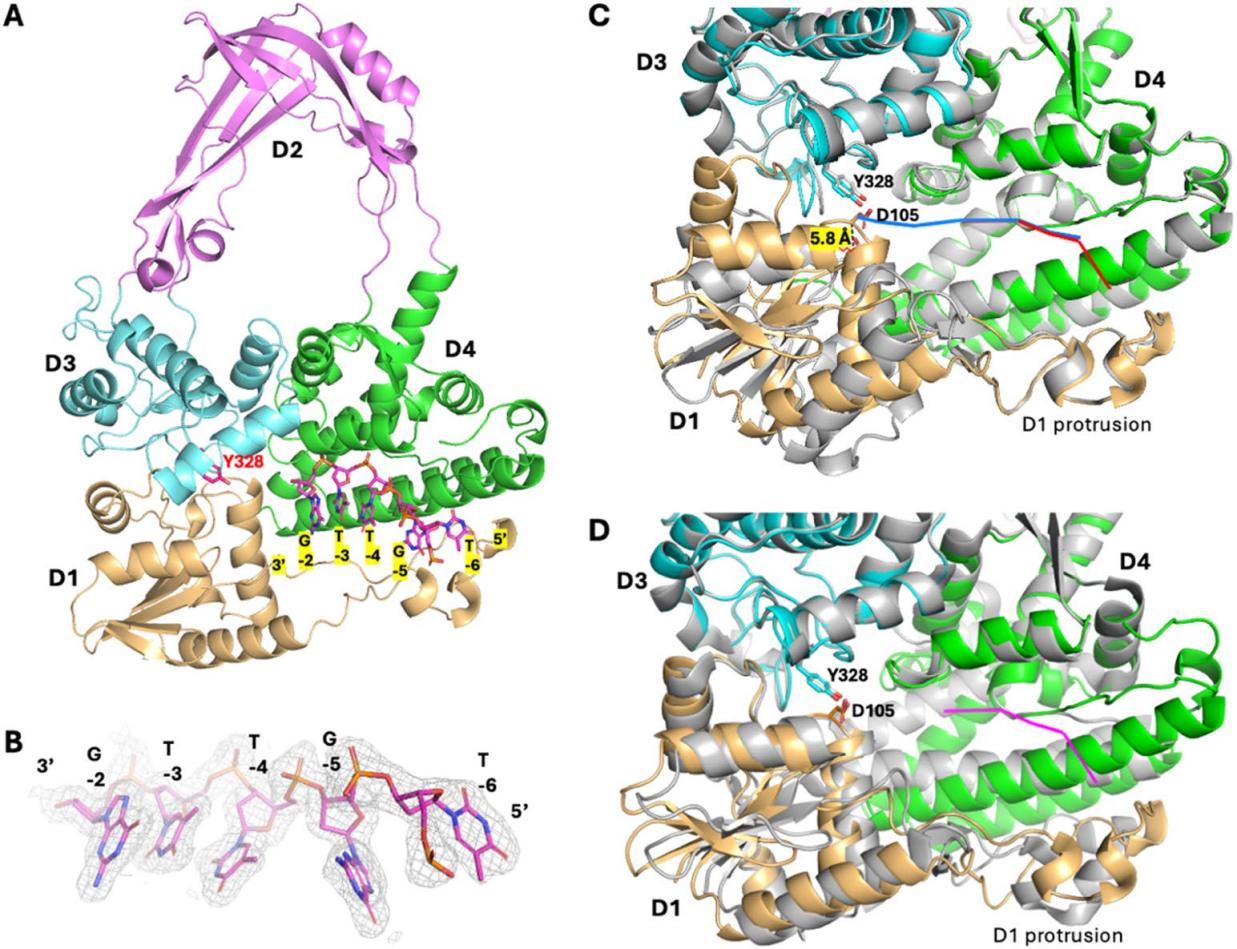
Half-open ssDNA-binding mode of EcTopo3. (**A**) Ribbon representation of the half-open binding mode in the EcTopo3–8MerD2 structure. The four domains, D1, D2, D3, and D4, are colored lime, pink, cyan, and green, respectively. Only the five 3′-terminal nucleotides of 8MerD2 (5′-AACTGTTG-3′) participate in binding to EcTopo3 within the D4/D1 binding groove. The three 5′-terminal nucleotides are expected to extend outside the binding groove and are presumed to be structurally disordered. The DNA oligonucleotide is shown in stick representation, and each nucleotide is labeled according to its binding position. The catalytic residue Y328 is highlighted to indicate its position relative to the bound oligonucleotide. (**B**) The light gray mesh shows the σA-weighted 2Fo–Fc electron-density map associated with the 8MerD2 ssDNA oligonucleotide, contoured at 1.0σ. No visible electron density can be assigned to the three 5′-terminal nucleotides of the 8-base oligonucleotide. (**C**) D4-based structural alignment of the open EcTopo3–8MerD1 structure (gray) and the half-open EcTopo3–8MerD2 structure (colored as in panel A). The catalytic residue Y328 and the active-site residue D105 from each structure are shown in stick representation to illustrate the interdomain conformational difference between D1 and D3 in the two structures. (**D**) D3-based structural alignment of the apo structure (PDB entry 1D6M; gray) and the half-open EcTopo3–8MerD2 structure (colored as in panel A). The DNA-binding site between the D3 and D1 domains is apparently closed in both structures.

An unexpected finding of this study is a previously unrecognized EcTopo3–ssDNA binding mode, represented by the EcTopo3–8MerD2 structure, in which EcTopo3 engages only the five 3′-terminal nucleotides of an 8-base oligonucleotide (Fig. 3A). The electron density for these five nucleotides is well defined at 1.90 Å resolution (Fig. 3B). Relative to the open EcTopo3–8MerD1 structure, the conformations of the D4 domain, the D1 protrusion, and the oligonucleotide backbone within the D4/D1 groove are largely unchanged despite differences in oligonucleotide sequence (Fig. 3C). The D3 domain also exhibits little conformational change, as judged by the position of the catalytic residue Y328.

In contrast, the main body of D1 in EcTopo3–8MerD2 is rotated by approximately 28.4° and shifted predominantly upward, with the active-site residue D105 displaced by about 5.8 Å. In the absence of nucleotides at the + 2, +1, and − 1 positions between D1 and D3, this movement of D1 fills the intervening space and closes the D1/D3 binding site. As a result, the relative arrangement of these two domains resembles that of the closed apo conformation^27^(Fig. 3D). Although D1 also shifts laterally outward relative to the apo structure, likely in response to oligonucleotide binding in the D4/D1 groove, the D1/D3 interface remains essentially closed. We therefore refer to this binding mode as ‘half-open,’ with the D4/D1 binding groove open and occupied by ssDNA, while the D1/D3 active-site region remains closed.

### Interaction patterns in half-open EcTopo3-ssDNA complexes

**Fig. 4:**
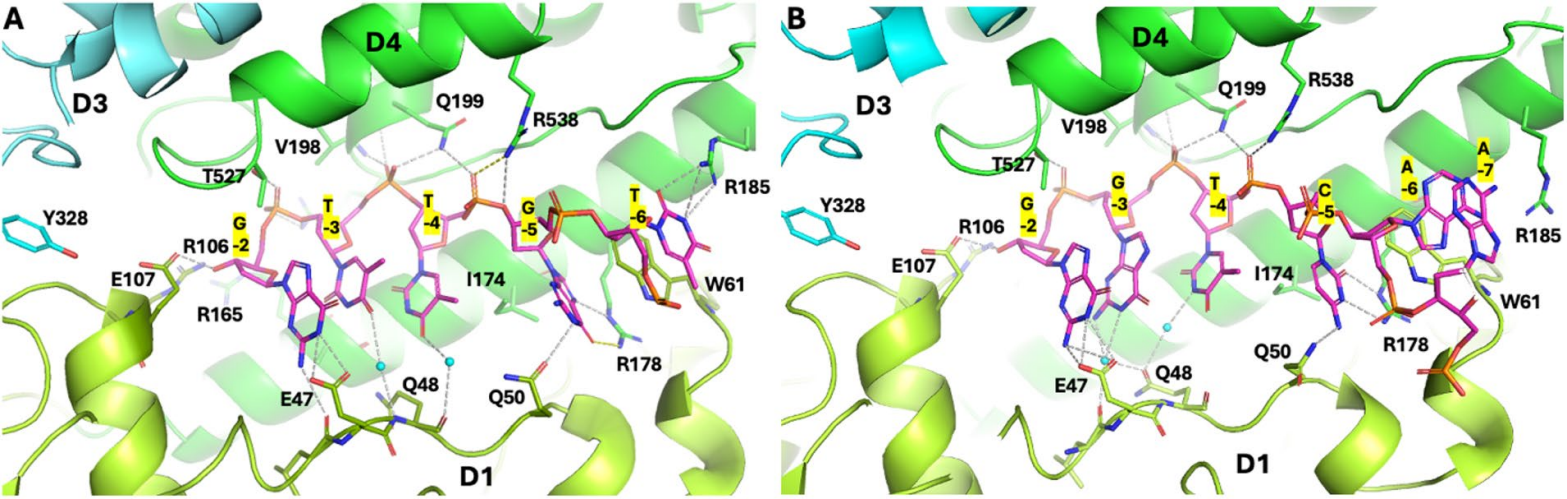
EcTopo3–oligonucleotide interactions in the half-open structures. (**A**) Interaction pattern in the D4/D1 binding groove of the EcTopo3–8MerD2 complex. A notable feature of this interaction pattern is the thymine at the − 3 position, which interacts with Q48 of the enzyme through a water-mediated hydrogen bond. Another thymine at the − 4 position is also connected to the enzyme through a water-mediated interaction. (**B**) Interaction pattern in the D4/D1 binding groove of the EcTopo3–8MerD3 complex. The major features of ssDNA binding in this second half-open structure, particularly at the 3′ end of the oligonucleotide, are similar to those observed in the open EcTopo3–8MerD7 structure (Fig. 2A).

In the co-crystallization of EcTopo3 with 8-base oligonucleotides, 8MerD2 and 8MerD3 (Table 1) each produced a half-open structure (Figs. 4). Structural superposition of these two structures by SSM yielded an RMSD of 0.51 Å, indicating that the conformations of the two half-open structures are nearly identical, even though the five 3′-terminal nucleotides bound in the D4/D1 binding groove differ in sequence (Fig. 4; Table 1). Interestingly, compared with the open structures, both half-open structures have higher resolution and lower B factors (Table 2), suggesting a potentially more rigid conformation.

Within the D4/D1 binding groove of the two half-open complexes, two residues from the D1 domain, R106 and E107, form hydrogen bonds with the ribose of the 3′-terminal guanine, apparently helping to fix the position of the oligonucleotide 3′ end. These two residues are part of the conserved DxDRE motif in D1, which is involved in catalytic divalent ion binding. In the open EcTopo3 complex, these residues help define the D1/D3 DNA-binding site mainly through van der Waals interactions, without forming specific protein–DNA hydrogen bonds.

The guanine base at the − 2 position forms multiple hydrogen bonds with E47 in D1. An additional water-mediated hydrogen-bonding network appears to further stabilize binding to this nucleotide. In the EcTopo3–8MerD2 structure (Fig. 4A), the nucleotide at the − 3 position is thymine. Because this smaller, single-ring pyrimidine base cannot make the same direct contacts as a purine, its interaction with the enzyme is mediated by a water molecule. A similar feature is observed for the pyrimidine nucleotide at the − 4 position in the open structures described above, as well as in the two half-open structures.

In the EcTopo3–8MerD3 complex (Fig. 4B), the principal interaction features at the 3′ end of the oligonucleotide are similar to those observed in the open EcTopo3–8MerD7 complex (Fig. 2A). With the exception of a G→A substitution at the − 3 position relative to EcTopo3–8MerD7, the interactions involving the − 4 to − 6 positions are largely similar. Again, R183 does not interact with the adenine at the − 6 position. Possibly because of strong base-stacking interactions between two adjacent adenines, the adenine at the − 7 position is resolved in this structure. As a result, only the two 5′-terminal nucleotides of the 8-base oligonucleotide are not visible in the structure and are presumed to be disordered outside the DNA-binding groove.

### A metal ion-binding site

**Fig. 5:**
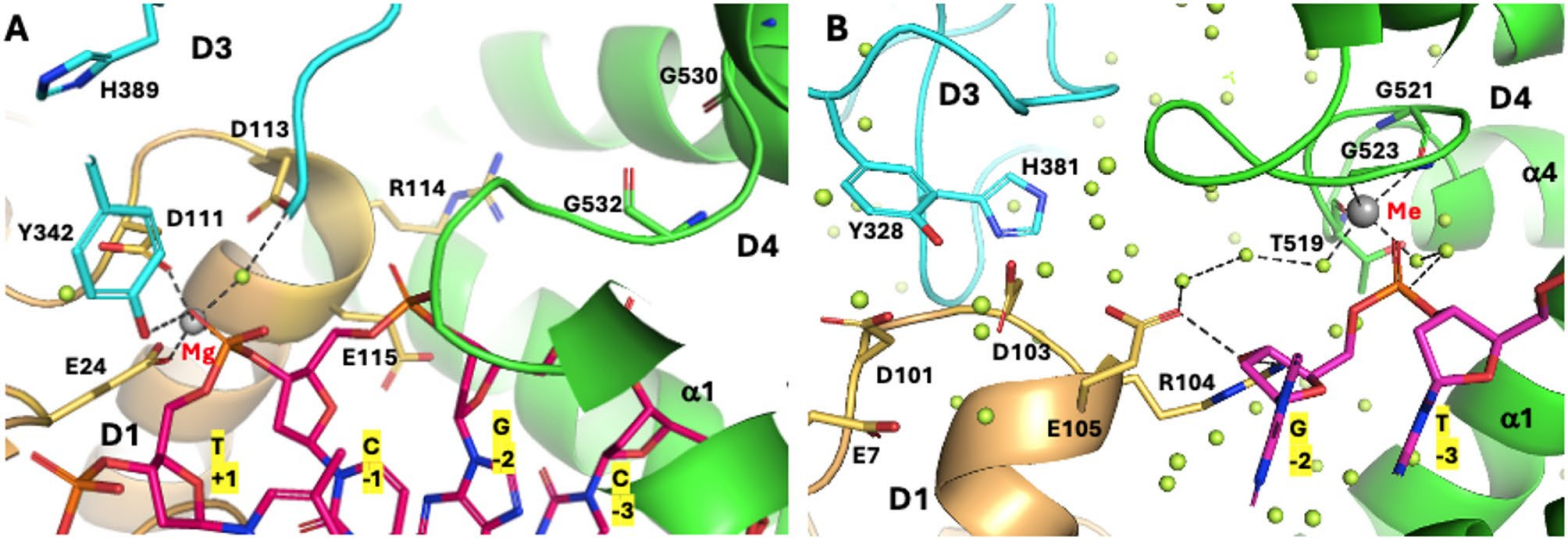
A metal-ion binding site proximal to the active site in the half-open structure. (**A**) Active site of the open-form MtbTopo1–MTS2-13 structure (PDB entry: 6CQ2). This structure represents a catalytically competent conformation. The three domains shown in this view, D1, D3, and D4, are colored light orange, cyan, and green, respectively. Water molecules and the metal ion are shown as lemon and gray spheres, respectively. Metal coordination bonds and a water-mediated hydrogen bond are indicated by black dashed lines. (**B**) Metal-ion binding site observed in the half-open EcTopo3–8MerD2 structure. In addition to the metal coordination bonds, selected water-mediated hydrogen bonds are shown to illustrate the proximity of the metal-binding site to active-site residues. Equivalent catalytic-site and metal-binding-site residues in MtbTopo1 and EcTopo3 are displayed as sticks for comparison. For clarity, the two structures are not shown in the same orientation.

Type IA topoisomerases function in an ATP-independent manner, requiring a divalent metal ion, typically Mg^2+^, for their catalytic activity. The magnesium ion plays a crucial role in the rejoining (religation) of the G-segment DNA. However, Mg^2+^ and other divalent metal ions have rarely been observed in structures of type IA topoisomerases. One exception is the MtbTopo1-ssDNA complex from *Mycobacterium tuberculosis* topoisomerase I (PDB entry 6CQ2; Fig. 5A)^33^. Divalent metal ions have also been identified in the apo structure of hTop3B^34^ and in one of its cryo-EM complex structures (35). In the active site of the 6CQ2 open-form structure, the Mg^2+^ ion is associated with the scissile phosphate, forming four coordination bonds: two to the phosphate group, two to catalytic-site residues, and one additional water-mediated hydrogen bond (Fig. 5A). No divalent ion has been identified in other EcTopo3, EcTopo1, MtbTopo1, or MsmTopo1 structures complexed with ssDNA. The sporadic observation of the metal ion in only one bacterial type IA topoisomerase structure suggests its transient nature.

Interestingly, in both half-open structures, EcTopo3–8MerD2 and EcTopo3–8MerD3, a metal-binding site was identified on the loop following the second helix (α4 of D4) after the EcTopo3 polypeptide chain returns to the D4 domain from the arch-like D2 domain, Fig. 5B. The metal ion is coordinated by the main-chain carbonyl oxygens of T519, G521, and G523, as well as by two conserved water molecules, in both high-resolution structures. The two coordinating water molecules bridge the metal ion to the DNA backbone and to active-site residues. The two glycine residues, G521 and G523, which form a GxG motif on the loop, are highly conserved. During structural refinement, the metal ion could be modeled as either Na⁺ or Mg²⁺, as these ions cannot be distinguished unambiguously in the electron-density map. Based on the average metal–ligand bond lengths, the ion was modeled as Na⁺ for refinement purposes. However, this assignment does not exclude the possibility that Mg²⁺ occupies the site, given the conformational plasticity of the glycine-rich loop.

No additional data are available regarding this metal-binding site or its possible connection to the scissile phosphate-associated metal-binding site shown in Fig. 5A. A metal-binding site near the active site could contribute indirectly to catalysis by stabilizing the local electrostatic environment, organizing catalytic residues, or facilitating substrate positioning. Its limited occurrence at different positions in available structures suggests a transient or conformation-dependent role during the reaction cycle^33–35^.

## Discussion

**Fig. 6:**
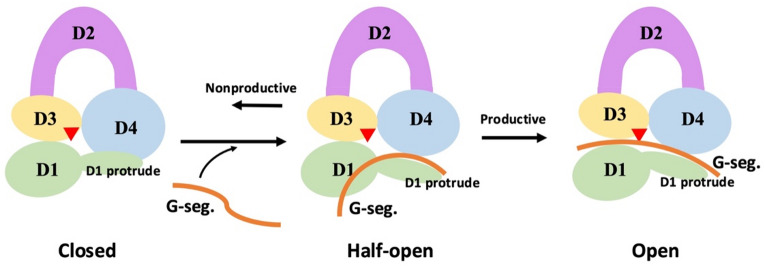
Schematic diagram of the proposed G-segment binding pathway. The half-open structure may represent a transient intermediate between the closed and open conformations. Depending on the G-segment sequence within the D4/D1 binding groove and other conditions, this transient state may be either productive or nonproductive. The D1 protrusion has been defined as part of D4 in some studies; in this case, D4 alone is responsible for forming the DNA-binding groove.

The five EcTopo3–ssDNA complex structures reported here provide a more nuanced view of ssDNA recognition by a type IA topoisomerase than was available from earlier works. Together, these structures suggest that EcTopo3 engages the G-segment through two structurally and functionally distinct regions: a highly conserved D4/D1 groove that accommodates much of the bound ssDNA backbone, and a more conformationally variable D1/D3 site positioned closer to the catalytic tyrosine. These data further suggest that occupancy of the D4/D1 groove does not require complete opening of the D1/D3 region, thereby revealing a previously unrecognized half-open binding mode that may represent a catalytically relevant intermediate along the DNA-binding pathway, although we cannot exclude the possibility that it is a nonproductive intermediate, Fig. 6. This conformation appears close to the intermediate state in which the D1/D3 interdomain space remains incompletely open, thereby preventing the nucleotides at the * -1*, * +1*, and * +2* positions from adopting the catalytically competent configuration^17^. A similar intermediate has also been reported in an *Ec*Topo1–ssDNA complex^36^. Additionally, the ssDNA oligonucleotide bound within the D4/D1 groove in half-open structure may represent the noncovalently bound cleavage product generated following G-segment cleavage, whereas the covalent 5′-phosphotyrosyl intermediate linked to the catalytic tyrosine contributes to opening of the gate for T-segment passage. This observation further supports the view that the principal role of the D1/D3 binding site is to ensure correct positioning of the G-segment for catalysis, rather than simply to promote G-segment binding. Such a model is consistent with the asymmetric binding of the G-segment in type IA topoisomerases.

Another important observation from this study is the strong structural conservation of the D4 domain and the ssDNA backbone within the D4/D1 binding groove across multiple complexes. Even when different 8-mer sequences are bound, the overall placement of the 5′ or 3′ portion of the oligonucleotide in this groove remains highly similar. This recurring feature supports the view that the D4/D1 groove serves as a stable anchoring site for the G-segment. At the same time, local base-specific interactions within this groove are not rigidly fixed. Sequence restriction, such as that adenine is disfavored at -5 position and to some extent at -6 position, is limited. Instead, the structures show that equivalent nucleotide positions can be recognized through either direct protein–DNA hydrogen bonds or water-mediated contacts. The comparison between the new EcTopo3–8MerD1 structure and the previously reported PDB entry 2O19 complex is very illustrative in this regard. In both structures, the overall binding geometry is similar, yet the nucleotides at the − 2 to − 4 positions engage the protein differently, largely due to different degrees of solvation. Thus, while the groove appears to impose a conserved backbone trajectory, it tolerates some variability in how individual bases are accommodated. With respect to possible nucleotide preferences at the + 2 to − 1 positions, smaller pyrimidine bases may be favored, given that the D1/D3 interdomain space must remain substantially open to allow these nucleotides to reach catalytically competent positions. Accommodation of smaller nucleotides would presumably require less conformational adjustment and, therefore, lower energetic cost. Consistent with this possibility, cytosine and/or thymine have been mostly observed at these positions in all reported open topoisomerase IA structures except an adenine in hTop3B complex^35^. Purine nucleotides at these positions may impose an unproductive conformation, in which their larger bases cannot be properly accommodated at the catalytic site^36^. In the co-crystallization experiments performed in this study, we were unable to obtain co-crystals with several of the 8-base oligonucleotides tested (Table 1). Owing to limitations in sample availability, it remains unclear whether this failure resulted from technical constraints of co-crystallization or from sequence-dependent effects that prevented formation of either the open or half-open conformations. Nevertheless, the observations described above raise the possibility that nucleotide sequence contributes to stabilization of these structural states. As for the specific failure to obtain EcTopo3/RNA cocrystals, one possible explanation is the difference in nucleotide packing between ssDNA and RNA. In topoisomerase IA complexes with ssDNA, the nucleotides adopt a largely B-form-like conformation, except at kinked positions. Such packing is unfavorable for RNA. Accordingly, a potential RNA-bound EcTopo3 complex would likely adopt a conformation distinct from those observed in the EcTopo3/DNA complexes presented here. It remains unclear whether such a conformation would be sufficiently stable or compatible with crystallization.

A further notable finding is the identification of a metal-binding site near, but distinct from, the active site. Although the current data do not establish a direct mechanistic link between this site and the scissile phosphate-associated metal observed in the MtbTopo1 structure (33), its location raises the possibility of an indirect catalytic or regulatory role. Metal binding near active sites can stabilize local electrostatics, help organize catalytic residues, or facilitate substrate positioning. The fact that metal binding is observed only in a limited structural context suggests that metal occupancy may be transient, conformation-dependent or potentially unrelated to catalytic activity. At the same time, the identity of the ion cannot be assigned with complete certainty from electron density alone, and the choice of Na⁺ during refinement does not exclude possible Mg²⁺ occupancy under appropriate conditions. Accordingly, any functional role for this site should be regarded as untested until supported by biochemical or mutational analysis.

## Data Availability

The crystal structures and associated structure factors generated in this study have been deposited in the Protein Data Bank under accession codes 11FC, 11GX, 11KB, 11LB and 11YY.
